# Topics of the Month

**Published:** 1896-02

**Authors:** 


### TOPICS OF THE MONTH.

The *Buffalo Morning Express*, our esteemed contemporary, has recently celebrated its semi-centennial anniversary. It was established January 15, 1846, by A. M. Clapp & Co., and has been a daily visitor at the residences of our citizens since that date. It issued a jubilee number January 15, 1896, consisting of fourteen pages, besides a facsimile of its first issue that was printed as a souvenir of the occasion. In the regular issue a number of columns were devoted to the history of the *Express* from its foundation to its golden anniversary.

The first paper was contributed by Hon. A. M. Clapp, of Washington, who for twenty-three years edited and owned the paper in whole or in part. This veteran journalist, still living and enjoying good health in the twilight of life, is a splendid specimen of stalwart manhood and forceful American character. His story of the founding and conduct of the *Express* during its early years is full of absorbing interest, especially to our older inhabitants who are more or less familiar with the events of that period.

This group of historical papers carries the *Express* from its beginning down to the present, tracing it through all the improvements in the printing art that have occurred during the last fifty years. It was the first paper in Buffalo to abandon the old blanket sheet and to adopt the present octavo form. It has kept pace with all other improvements and is now printed on the most modern presses, is set by type machines and is in every way a foremost newspaper.

In the souvenir facsimile the advertisements constitute a curiously interesting study. We learn through them how frequently and rapidly one could go from Buffalo to New York, there being one train a day. Further, that a Wells & Co.'s express messenger was sent out from the office, No. 1 Exchange street, on Saturday of each week for Cleveland, Columbus, Chicago, and othes cities in the West. This messenger was presumably Mr. Fargo himself.

An interesting advertisement relating to the founder and first editor of the JOURNAL we reproduce in full :

☞ YOUNG MEN'S ASSOCIATION LECTURES.—Notice is hereby given to the members and the public that an arrangement has been concluded with AUSTIN FLINT, M. D., of this city, in pursuance of which he will deliver a course of Lectures upon Anatomy, illustrated by means of a Mannikin, provided a sufficient number of subscribers can be obtained.

The proceeds of these Lectures will be expended for the purchase of a Mannikin, to be used in illustration of the course, and which is to be retained as the property of the Association.

Teachers of Academies and Public Schools, who may wish to attend these lectures with their pupils, will be supplied with tickets at reduced prices upon application to the Lecture Committee.

Subscriptions will be received at the different Book-stores, and by the Executive Committee of the Association.

☞ Single tickets to the course $1. Ticket to admit a Lady and Gentleman $1.50. Family Tickets $2.50.

TH. C. WELCH,
JAS. SHELDON, JR.,
N. K. HOPKINS,
JNO. M. HUTCHINSON,
S. B. HUNT,
Lecture Committee.

Our readers will observe that Dr. Flint was a man of versatile talent, who devoted much of his time to disseminating information among the people.

We venture to offer our congratulations to the *Express*, whose present owners are easily making it one of the foremost American newspapers. We trust the JOURNAL may enjoy the felicitous opportunity of congratulating the *Express* when it shall celebrate its centennial anniversary.

---

THE water supply of a great city should ever receive the most jealous care and be kept from even the remotest danger of contamination. Formerly in Buffalo we boasted of the purest and most perfect system ; but of late our water supply is open to the suspicion of contamination by sewage. This, to be sure, is denied by many microscopists, who affirm that as yet no organic matter has been discovered containing typhoid or other noxious germs. Be this as it may, the Niagara water is in general so roily as to be offensive to the eye and is not easily disassociated in thought from dirt. This should not be so. It is all very well to direct the inhabitants individually to filter water to be used for drinking purposes, but very few will comply with such advice. The fact is, the city itself should do the filtering and furnish to consumers water not only free from diseased germ contamination but that which presents to the eye a clear and attractive appearance. The more wholesome the water the less beer will be drunk.

---

CREMATION appears to be growing in favor in this country. According to the *Tribune Almanac* there are twenty-six associations in active operation in the United States. The oldest was organised at Washington, Pa., in 1876, and the two newest were found at New Haven, Conn., and Elizabeth, N. J., in 1894. The number of incinerations is reported as 3,670. The number incinerated in Europe from 1876 to 1893 was 19,700. The membership of the American associations is about 8,000 and the adherents of the method about 100,000.

---

THE Craig Colony, a home for the epileptics located at Sonyea, Livingston County, N. Y., was opened for the reception of patients Monday, January 20, 1896. It had been determined that the number of patients first to be received should be limited to thirty.

They were taken from the counties of the state pro rata under and by direction of Dr. Charles S. Hoyt, secretary of the State Board of Charities. From time to time other epileptics will be admitted until the limit of the colony accommodations is reached, which is 200.

The formal opening ceremonies will take place in June, 1896. One object in receiving a limited number at first is to employ each at the occupation which suits him best, determined though after a careful examination by the colony physicians. The aim has been to select farmers and gardeners in the first group.

Masons, carpenters, painters and shoemakers will also be received among the first patients. The resources of the land at Sonyea are such that in one year from date it is expected the colony will be self-supporting, so that even from the viewpoint of economy the managers say the scheme is a wise one.

It is expected that in time people of means, impressed by the worthiness of the charity, will liberally endow the colony and its various industrious schools. Villas bearing the names of the donors will doubtless spring up. Some wealthy people also will build cottages for afflicted members of their own families in the parks which abound. It is hoped that the colony physicians and pathologists may some day discover the causes and cure of epilepsy. The object of Craig Colony will, as its managers state, be fourfold: to provide schools where epileptics may attain any degree of education; to provide industrial training of all kinds, there being no vocation which some epileptics may not follow; to give them homes when all other doors are closed against them, and to see that each and every case is carefully studied and treated in accordance with the best scientific methods the world affords.

Applications have been received from foreign countries—Canada, South America, Russia and Asia. Those who have helped to create a sentiment which has resulted in the establishment in this state of possibly the finest colony for epileptics in the world, and those who have aided the establishment in any way will feel some degree of pride in knowing that their efforts have been crowned with great success. The Craig Colony is one of the most philanthropic institutions in this state and in every way commands the respect of a generous and sympathetic people.

The board of managers has made an appeal to all interested to contribute toward the formation of a library for the use of patients. Nearly every one has spare copies of good books—biographies, histories, poetry, fiction, bound volumes of magazines or something suitable for such a purpose. It would add to the value of the collection if each donor should write his or her name upon the fly-leaf of the book. The books or money can be forwarded by mail or express to Dr. William P. Spratling, superintendent, Sonyea,

Livingston County, N. Y., or if from the 8th Judicial district, to Miss Edna Oatman, No. 388 Grant street, Buffalo, N. Y., and correspondence directed to her will be answered if any organisation wishes to know more regarding the wants of the institution. The members of the board of managers from this judicial district are William H. Cuddeback and Leroy S. Oatman, either of whom will gladly give information to any, or will take care of any donations sent to them or to Miss Oatman, as above directed.

---

Spitting in public places has become not only an intolerable nuisance, but a menace to health. It is well known that germs from dried sputum mixed with atmospheric air are easily respired, thus making consumption a communicable disease. The Board of Health of the city of New York, it is reported, will soon take action upon a report made by Dr. Hermann M. Biggs, pathologist, and Dr. T. Mitchell Prudden, consulting pathologist, to the board. The report asseverates that it is time to put a stop to the filthy and disgusting practice of spitting on the floors of public buildings and conveyances. The report declares that it has long since been shown that the chief means for the transmission of consumption is the dried and pulverised sputum of persons suffering from this disease. Diphtheria, influenza and other diseases, the report says, are also easily communicated in this way during certain stages of the diseases. Catarrhal affections may be communicated through dried spittle mixed with dust. These germs are likely to be gathered on the feet and on the skirts of women, the report says, and taken into private houses, where the most perfect ventilation will not stay their evil effect.

The report recommends the adoption of the following resolutions:

*Resolved,* That notices be posted in all public places and in all surface and elevated cars in this city, signed by the Board of Health, warning passengers against expectoration upon the floors of these conveyances, and further, that similar notices be posted in the stations of the elevated roads, warning against expectoration upon the platforms and stairs or on the floors of the stations.

*Resolved,* That similar notices be posted in the halls and assembly rooms of all municipal and federal buildings in the city.

*Resolved,* That the municipal authorities be requested to provide sufficient and proper receptacles for expectoration for such public places as are in their control, and that the managers of the elevated roads be requested to provide similar receptacles sufficient in number for their stations and platforms, and that in all cases these receptacles shall be kept in a cleanly condition.

*Resolved*, That the officers of the Manhattan elevated road be requested to give peremptory orders to their guards to refrain from, and to prevent so far as possible, expectorations from the trains into the streets, and to secure the enforcement of these orders.

In Buffalo, notices are posted in the street cars requesting passengers not to spit upon the floor, but until conductors are empowered with special police authority to enforce the regulation it is practically a dead letter. We think the time has arrived for the board of health to take such action as will prevent spitting in public places.

---

The Consolidated Library of New York, consisting of the Astor, Lenox and Tilden libraries, has secured the services as librarian of Dr. John S. Billings, director of the Department of Hygiene of the University of Pennsylvania. He is considered the best bibliographer in the United States, and as the consolidated library will be the largest in this country, having about 374,000 volumes and property and endowments amounting to about $8,000,000, it is fitting that he should be appointed.

---

Dr. Ernest Wende, health commissioner, deserves the thanks not only of every physician but of every respectable citizen for the steps he has taken to prevent the so-called Christian scientists from practising medicine in Buffalo. It is affirmed, in the newspapers, that he intends to invoke all the power that the law arms him with to suppress this dangerous nuisance.

The first practical step made to stamp out this practice was taken Thursday, January 9, 1896, when Dr. Wende and Health Inspector Willard of the health department appeared before the grand jury and gave evidence against Mrs. Carrie Jenner, a so-called Christian scientist, who lives at Black Rock. The health commissioner and his assistants charge her with practising medicine illegally, inasmuch as she has no license to prescribe any medicine, no matter how simple.

It is stated that the district attorney, Mr. Kenefick, is lending all the influence and skill of his office to aid Dr. Wende in his commendable efforts to enforce the law against a class of pretenders, all the more dangerous because they appear so childlike and bland

The *Post-Graduate*, in its issue for January, makes some pertinent suggestions regarding needed medical legislation. We need not go into the matter in detail, preferring to let the *Post-Graduate*, which is perfectly competent to do so, speak for itself as follows:

We find that the Chairman of the Committee on Legislation of the Medical Society of the State of New York is a member of the Post-Graduate Faculty. He being, therefore, one of our own family, we beg to suggest very plainly to him what ought to be done during the coming winter.

First, the obnoxious law which prevents a doctor from being president of the (New York City) board of health, should be repealed. Second, the anatomy bill, which has passed so many legislatures, and has, under one or another pretext, never reached the Governor, or has been returned, or has been vetoed by him, should be looked after from start to finish. On the first subject, we have no doubt, the *Post-Graduate* is in accord with every member of the profession in the state. We cannot imagine a doctor in medicine, who thinks that his profession ought to be made a subject of special legislation, when that special legislation is to exclude him from a post of honor and influence. It is a reflection upon us as a body which no self-respecting man can condone. One of the best presidents the Board of Health ever had was the late Dr. Willard Parker—indeed, we believe he was its first president.

---

The *Comité Franco-Américain*, of Paris, to which we made reference in the January issue of the Journal, concerning the promotion of the interests of American students in France, we learn has made arrangements with La Companie Transatlantique whereby, in accordance with the recommendation of the committee, a reduction of 30 per cent. from the price of the passage between New York and Havre will be made to such American students as propose to study under a faculty in France. Application should be made to M. Paul Melon, 24 place Malesherbes, Paris.

